# Supplementary material for: Sulfur-Doped Organosilica Nanodots as a Universal Sensor for Ultrafast Live/Dead Cell Discrimination
Source: Biosensors (Basel). 2022 Nov 10;12(11):1000. doi: 10.3390/bios12111000 (PMC9688158; doi:10.3390/bios12111000)
Supplement: Supplementary file 1 [file biosensors-12-01000-s001.zip › biosensors-1969242-supplementary.pdf]

Supporting Information

Sulfur-Doped Organosilica Nanodots as a Universal Sensor for Ultrafast Live/Dead Cell Discrimination

Yan-Hong Li†, Jia Zeng†, Zihao Wang, Tian-Yu Wang, Shun-Yu Wu, Xiao-Yu Zhu, Xinping Zhang, Bai-Hui Shan, Cheng-Zhe Gao, Shi-Hao Wang and Fu-Gen Wu*

State Key Laboratory of Bioelectronics, School of Biological Science and Medical Engineering, Southeast University, 2 Sipailou Road, Nanjing 210096, China

Corresponding Author

*E-mail: wufg@seu.edu.cn (F.G.W.)

Author Contributions

†These authors contributed equally to this work.

Materials

Escherichia coli (*E. coli*), *Staphylococcus aureus* (*S. aureus*), and *Saccharomyces cerevisiae* (*S. cerevisiae*) yeast were obtained from the China Center of Industrial Culture Collection (CICC, Beijing, China). HPAEpiCs (a human alveolar epithelial cell line) were bought from GuangZhou Jennio Biotech Co., Ltd. (China). A549 (a human lung cancer cell line) cells were purchased from Wuhan Servicebio Technology Co., Ltd. (China). Urea, dimethyl sulfoxide (DMSO), *N*,*N*-dimethylformamide (DMF), ethanol, and citric acid monohydrate were bought from Sinopharm Chemical Reagent Co., Ltd. (Shanghai, China). Lysogeny broth (LB), potato dextrose water (PDW), and PDW agar were obtained from Beijing Land Bridge Technology (Beijing, China). Bis[3-(triethoxysilyl)propyl]tetrasulfide was purchased from Heowns Biochem Technologies (Tianjin, China). 3-(4,5-Dimethyl-2-thiazolyl)-2,5-diphenyl-2-*H*-tetrazolium bromide (MTT) was bought from J&K Scientific Ltd (Beijing, China). RedDot™2 Far-Red Nuclear Stain (RedDot2) was brought from Biotium, Inc. (Hayward, USA). Dialysis membranes (regenerated cellulose, molecular weight cut-off (MWCO): 100 Da) were from SpectrumLabs, Inc. (Rancho Dominguez, CA). All solutions/suspensions were prepared with deionized water (18.2 MΩ•cm) obtained by a Milli-Q water purification system (Millipore, Billerica, MA).

Synthesis of orange-emitting sulfur-doped organosilica nanodots (S-OSiNDs)

The S-OSiNDs were prepared according to our previous study [1] (Note that in our previous work [1], the concentrations of the raw materials bis[3-(triethoxysilyl)propyl]tetrasulfide, citric acid, and urea, which were dissolved in DMF, were wrongly expressed as 0.4, 3, and 2 mg/mL, respectively; the correct concentrations of the three raw materials should be 0.04, 0.3, and 0.2 M, respectively.). Briefly, 5 mL DMF solution containing bis[3-(triethoxysilyl)propyl]tetrasulfide (0.04 M), citric acid monohydrate (0.3 M), and urea (0.2 M) via a solvothermal method (200 ^{o}C, 12 h). The obtained S-OSiNDs were further purified via 6 h dialysis against pure water with a dialysis membrane (MWCO: 100 Da). Then the as-prepared S-OSiNDs

were stored at 4 °C for further use.

Characterization

Transmission electron microscopy (TEM) images were obtained from an FEI Tecnai G2 20 transmission electron microscope. Ultraviolet–visible (UV–vis) and fluorescence spectra were collected on a UV–vis spectrophotometer (UV-2600, Shimadzu, Japan) and a spectrofluorophotometer (RF-5301PC, Shimadzu, Japan), respectively. The infrared spectra were recorded with a Fourier transform infrared (FTIR) spectrometer (Nicolet iS50, Thermo Scientific, USA). X-ray photoelectron spectroscopy (XPS) experiments were carried out by a PHI Quantera II X-ray photoelectron spectrometer (ULVAC-PHI, Inc., Japan). Flow cytometric experiments were performed using a flow cytometer (NovoCyte 2070R, ACEA Biosciences Inc., USA).

Cell culture and preparation of live/dead cells

Bacterial cells including *E. coli* and *S. aureus* were separately cultured in LB media in a shaking incubator (180 rpm) at 37 °C. *S. cerevisiae* yeast cells were cultured in PDW medium in an incubator at 28 °C. HPAEpiCs and A549 cells were cultured in Dulbecco's modified Eagle's medium (DMEM) with 10% fetal bovine serum (FBS), supplemented with 100 U/mL streptomycin and 100 U/mL penicillin at 37 °C and 5% CO_2.

Toxicity evaluation of S-OSiNDs toward bacterial and mammalian cells

We carried out additional toxicity evaluations of S-OSiNDs toward *S. aureus*, *E. coli*, and yeast cells. Since the number of bacterial cells is well related to the turbidity of the bacterial suspension, the cytotoxicity of S-OSiNDs to *S. aureus* and *E. coli* was measured by recording the optical density at 600 nm (OD_{600}) via the microplate assay. First, different concentrations of S-OSiND suspensions (300 μL) were prepared (0, 10, 20, 40, 100, 200, 400, and 1000 μg/mL). Then they were separately mixed with the diluted suspensions of *S. aureus* and *E. coli* with the same volume of 300 μL. Finally,

the cells were seeded into each well of the 96-well plates. During the 24 h incubation process under shaking at 37 °C, the OD_{600} value of each well was recorded at different time points.

Since yeast cells were easily deposited onto the bottom of the container during the growth process, the cytotoxicity of S-OSiNDs toward yeast cells was measured using the colony forming unit (CFU) counting method, instead of the above-mentioned OD_{600} measurement. In brief, the log-phase yeast cells were cultured in PDW media containing different concentrations of S-OSiNDs (0, 5, 10, 20, 50, 100, 200, and 500 μg/mL) at 28 °C for 24 h. Afterwards, the diluted fungal suspensions (100 μL) at a dilution of 1:1000 were then plated separately onto PDW agar plates. After incubation at 28 °C for 24 h, the PDW agar plates were imaged using a digital camera and the number of the bacterial colonies formed on these plates was counted manually.

HPAEpiCs and A549 cells were cultured in a 96-well plate (5×10^3cells/well) for 12 h and treated with different concentrations of S-OSiNDs (0, 5, 10, 20, 50, 100, 200, and 500 μg/mL) for 24 h. Afterwards, 10 μL of MTT (5 mg/mL) was added to each well and incubated with the cells for 6 h. Next, 150 μL of DMSO was added to each well and mixed by shaking. Finally, the absorbance at 492 nm was measured using a Multiskan FC microplate reader (Thermo Scientific, USA). The cell viability was calculated by the following formula: Cell viability (%) = (mean absorbance of treated group/mean absorbance of untreated group) × 100%.

Supplementary figure

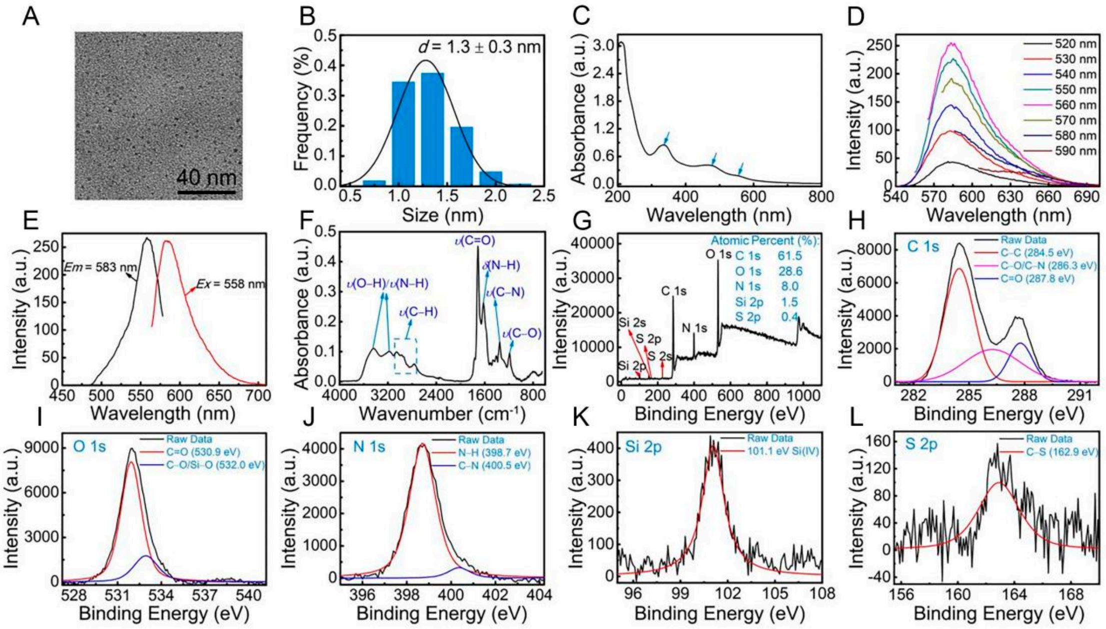

Figure S1. Characterizations of S-OSiNDs. (A) TEM image and (B) corresponding size distribution result. (C) UV–vis spectrum. (D) Fluorescence (FL) emission spectra collected at various excitation wavelengths and (E) maximum excitation and emission spectra. (F) FTIR spectrum. (G) XPS pattern of S-OSiNDs. (H–I) High-resolution XPS peaks of C 1s, O 1s, N 1s, Si 2p, and S 2p, respectively. Reproduced with permission from [1]. Copyright 2021, American Chemical Society.

Reference

1. Zeng, J.; Hua, X.W.; Bao, Y.W.; Wu, F.G. Orange-emissive sulfur-doped organosilica nanodots for metal ion/glutathione detection and normal/cancer cell identification. *ACS Appl*. *Nano Mater*. **2021**, *4*, 6083−6092.
